# Structure Design and Characterization of 3D Printing System of Thermal Battery Electrode Ink Film

**DOI:** 10.3390/mi14061147

**Published:** 2023-05-29

**Authors:** Fengli Liu, Jiale Lu, Yongping Hao, Yao Chang, Kuaikuai Yu, Shuangjie Liu, Zhiwei Chu

**Affiliations:** 1 School of Mechanical Engineering, Shenyang Ligong University, Shenyang 110159, China; 1836244593@163.com (J.L.); yphaosit@126.com (Y.H.); a202315541074724@163.com (Z.C.); 2 National Key Laboratory of Electromagnetic Space Security, Tianjing 300308, China; cjing1129@126.com (Y.C.); kuaikuaihit@126.com (K.Y.); 3 School of Equipment Engineering, Shenyang Ligong University, Shenyang 110159, China

**Keywords:** droplet ejection, piezoelectric micronozzle, 3D print system

## Abstract

In this paper, a 3D printing system for a thermal battery electrode ink film is set up and investigated based on the on-demand microdroplet ejection technology. The optimal structural dimensions of the spray chamber and metal membrane of the micronozzle are determined via simulation analysis. The workflow and functional requirements of the printing system are set up. The printing system includes a pretreatment system, piezoelectric micronozzle, motion control system, piezoelectric drive system, sealing system, and liquid conveying system. Different printing parameters are compared to obtain optimized printing parameters, which can be attributed to the optimal pattern of the film. The feasibility and controllability of 3D printing methods are verified by printing tests. The size and output speed of the droplets can be controlled by adjusting the amplitude and frequency of the driving waveform acting on the piezoelectric actuator. So, the required shape and thickness of the film can be achieved. An ink film in terms of nozzle diameter = 0.6 mm, printing height = 8 mm, wiring width = 1 mm, input voltage = 3 V and square wave signal frequency = 35 Hz can be achieved. The electrochemical performance of thin-film electrodes is crucial in thermal batteries. The voltage of the thermal battery reaches its peak and tends to flatten out at around 100 s when using this printed film. The electrical performance of the thermal batteries using the printed thin films is found to be stable. This stabilized voltage makes it applicable to thermal batteries.

## 1. Introduction

The thermal battery is an important military battery due to its high energy density and power density, wide ambient temperatures, long storage time, fast and reliable activation, compact structure, simple process, low cost, no need for maintenance, etc. [1]. The traditional method involves pressing the cathode, separator and anode powder layer by layer into a pellet [2]. The energy density of the thermal battery decreases due to the thickness of the electrode pellet. A strong interfacial stress appears between the cathode and electrolyte. The larger area electrode is difficult to press. It is difficult to control the electrode material, which results in an uneven film. Film electrodes can overcome the above shortcomings, which is why they attract attention. Film electrodes have been widely used in lithium-ion [3] and sodium-ion batteries [4]. The current main methods of preparing electrode film include radio frequency sputtering, pulsed laser deposition, electron beam evaporation, hydrothermal methods, metal-organic chemical vapor deposition, electrostatic spray deposition methods, sol-gel and coating methods, etc. These traditional methods have high equipment requirements, complex operations and slow production speeds. The main drawbacks are the difficulty in accurately controlling the film shape and scale, as well as the need for subsequent high-temperature heat treatment [5].

The fabrication of film electrodes of thermal batteries has also been investigated by using different methods recently. As one of the major methods, ink-jet printing technology has obvious advantages and characteristics. Ink-jet printing technology as additive manufacturing has recently been applied in bioengineering, micro-electro-mechanical systems (MEMS), optical engineering and other fields, especially in microdevice packaging and printing, in order to achieve accurate, efficient and stable distribution of microdroplets [6]. Ink-jet printing technology mainly includes continuous injection (CIJ) and on-demand injection (DOD). An on-demand ink jet has the characteristics of fast response and easy control compared to a continuous ink jet [7]. Li G et al. provided an in-depth and clear understanding of the high-viscosity droplet formation and also lays the foundation for the morphological control of conductive lines in high-viscosity paste DOD printing [8]. P. Masset et al. proposed thin-film cathodes prepared by a thermal-spray process [9]. Hu et al. employed screen-printing to construct a 50 µm thin-film cathode [1]. Both methods need a tedious preparation process. The high cost and complex equipment limit their applications. Kai Lia designed a piezoelectric micronozzle in which a tin microdot diameter of 340 µm can be printed on the surface when a voltage pulse with an amplitude of 16 V is applied to the designed piezoelectric nozzle [10].

In this paper, 3D printed electrode film is used to solve the influence of the interfacial stress. The printed thickness is less than the compressed thickness, which results in increasing the energy density. Large-area uniform electrodes can be obtained by adjusting the drive frequency, amplitude and speed. In order to simplify the equipment, a simple piezoelectric ceramic driver with a lever amplifier reduces the cost and increases the efficiency. The magnification of the piezoelectric lever amplification structure increases to 26 times, and the maximum output displacement reaches 790 μm with a drive voltage amplitude of only 3 V. The injection performance is simulated according to its flow field in different cross-section spray nozzle cavities. A square wave is chosen to drive the piezoelectric stack compared to the sina and triangle waves. The influences of the drive waveform amplitude, frequency, nozzle height and diameter on the solidification shape and dimensions of the printed lines are discussed. The speed, diameter and frequency of the jet can be controlled by adjusting the waveform parameters of the square wave at any time to realize on-demand injection. The influence of the metal diaphragm’s diameter on the frequency characteristics is studied. Based on the optimized parameters, a 3D printing system is manufactured with the corresponding workflow. The printing material is uniformly printed on the substrate due to the precision control of the piezoelectric ceramic driver. A film with a 1 mm wiring width and 0.1 mm thickness can be printed on the substrate with a signal frequency of 35 Hz when a voltage pulse with an amplitude of only 3 V is applied to the designed piezoelectric stack with a nozzle diameter of 0.6 mm at 90 mm/s nozzle movement speed. At the same time, material waste is reduced. The stable voltage is higher than that of the traditional method. In this printing system, the device has the advantages of high precision, fast response, high efficiency, easy operation and low drive voltage. The discharge performance is tested, which proves the stability of the proposed thin-film printing method. This work can also be applied to other substances such as pure glycerol liquid.

## 2. Design and Simulation of Spray Nozzle Cavity Structure

### 2.1. Design Principle of Volumetric Piezoelectric Nozzle

Two basic conditions are required for the volumetric piezoelectric micronozzle to realize the microdroplet ejection [11]:

(1) Overcoming the surface free energy effect of the liquid at the nozzle end face.

The relationship between the Weber number *We* and nozzle size parameters can be obtained as follows:

(1) $${}_{W_{e}\geq \frac{2}{3}\frac{pr_{1}{}^{2}}{\delta t_{r}}}$$

where *p* and *δ* are the pressure in the chamber and the coefficient of ink surface tension, respectively;$\;r_{1}$ is the diameter of the nozzle; $t_{r}$ is the length of the nozzle.

(2) The volume of the ejected microdroplets is larger than the critical volume.

The droplet volume $V_{jet}$ of the nozzle can be obtained using condition ②, which satisfies the following relationship:

(2) $$V_{jet}\geq \frac{1}{6}\pi r_{1}{}^{3}$$

It can be seen from Equations (1) and (2) that the injection conditions for the change of diaphragm amplitude can be described by the following equation:

(3) $$\Delta l=\frac{3}{2}\frac{W_{e}}{K_{v}}(\frac{t_{r}}{r_{1}})(\frac{h\delta }{r_{1}}K_{s})$$

where $K_{v}$ is the volume coefficient of the liquid, which is approximately constant at about 0.3; h is the height of the spray cavity$;\;K_{v}$ is the volume compressibility coefficient of the liquid.

### 2.2. Establishment of Basic Structure of Spray Nozzle Cavity

**Figure 1:**
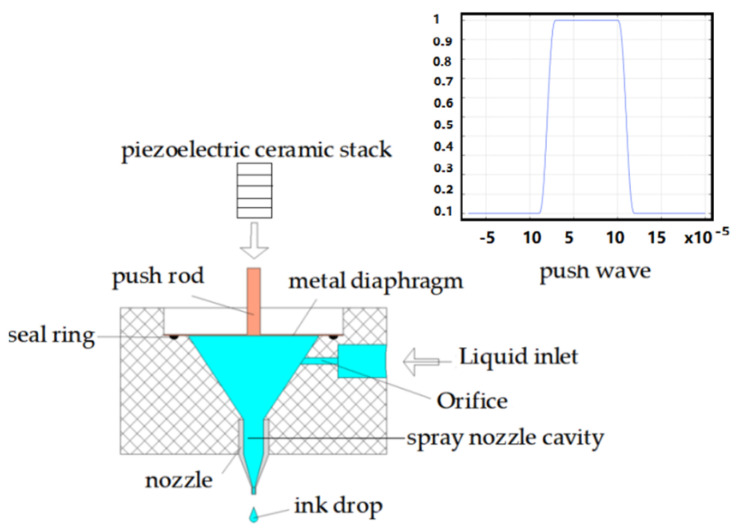
Schematic of the volumetric piezoelectric spray nozzle.

Piezoelectric stack actuator has the advantages of simple structure, fast response and high driving accuracy [12,13]. A diagram of the volumetric piezoelectric micronozzle design is shown in Figure 1. 

**Figure 2:**
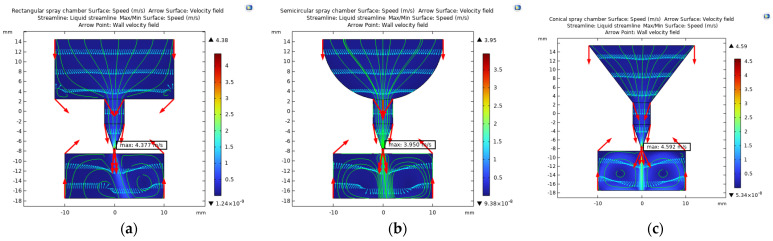
Velocity nephogram of spray nozzle cavity. (**a**) Flow velocity nephogram of rectangular section. (**b**) Flow velocity nephogram of semicircular section. (**c**) Flow velocity nephogram of conical section.

The generated vibrations are transmitted to the pushing rod via a lever displacement amplifier when voltage pulses are applied to the piezoelectric actuator. Then, the pushing rod drives the diaphragm to generate pressure waves in the cavity. The printing characteristics of the three types of nozzle cavities with rectangular, semicircle and conical cross sections are compared under the same input conditions. The boundary condition is set at a speed of 0.05 m/s. The density is 1000 kg/mm^3^, the dynamic viscosity is 50 cps, and the initialization speed is γ = 0.1 mm/s, the laminar flow wall condition is slip, the grid is divided according to the hydrodynamics, and the boundary layer is set to eight layers. The velocity nephogram of the spray nozzle cavities is shown in Figure 2.

The red arrow line indicates the change in the velocity direction of the fluid at the cavity boundary, the green arrow line indicates the flow field, and the blue arrow line indicates the fluid velocity field. It can be seen from the velocity cloud diagram that the output speed of the rectangular section spray nozzle cavity is 4.38 m/s. It can be seen from the flow field lines that the vortexes are formed at the lower end face and vertical wall face of the spray nozzle cavity at a high speed, which hinders fluid transmission. The maximum output speed of the semicircular section is 3.96 m/s, and the injection performance of the spray nozzle cavity is poor. This may be because the fluid conflicts with the incoming liquid under the effect of inertia and offset part of the kinetic energy due to the small upswept radian of the upper-end surface when the fluid flows to the upper end of the nozzle through the semicircular wall surface. The maximum output velocity of the conical spray nozzle cavity is 4.78 m/s because the flow channel of the chamber section has a gradient shape, which reduces the loss of fluid kinetic energy and the spray performance is optimal.

**Figure 3:**
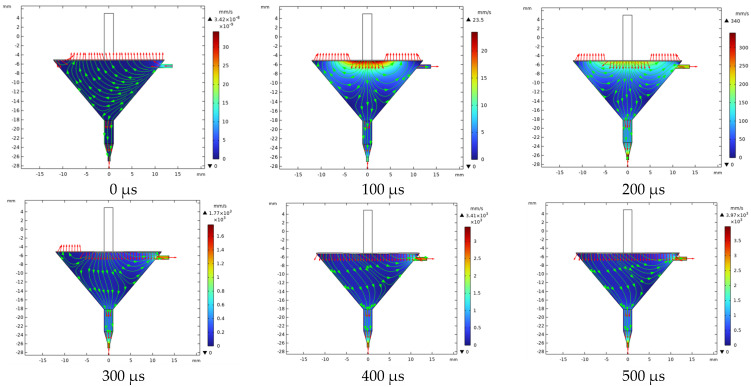
Fluid–solid coupling analysis of the piezoelectric micronozzle.

The fluid–solid coupling analysis of the piezoelectric micronozzle is shown in Figure 3.

The fluid enters the cavity through the inlet before the nozzle starts working at 0 s. The diaphragm is subjected to pressure from the fluid after filling the cavity and the pressure contour line is denser at the inlet, while it is more sparse away from the inlet. When the time reaches 100 μs, the membrane begins to deform downwards under the force. The fluid below the push rod begins to change its flow direction and gradually spreads around in the form of “water ripples”. The pressure is mainly concentrated in the area where the velocity direction begins to change in the pressure field, which is sparse in the surrounding areas with a dense trend in the middle. Moreover, the squeezed fluid also flows toward the inlet and nozzle. If the resistance at the inlet is small, more fluid flows back along the inlet. When the time reaches 500 μs, the membrane deforms to reach its maximum value, and the direction of fluid movement below the membrane is basically opposite to the initial state, completing the empowerment. At this time, the maximum speed is 3.97 mm/s. When the time reaches 600 μs, the diaphragm begins to recover, and the fluid begins to change in the velocity direction from around.

## 3. Design and Analysis of Vibrating Metal Diaphragm

### Analysis of the Size Parameters of the Metal Diaphragm

**Figure 4:**
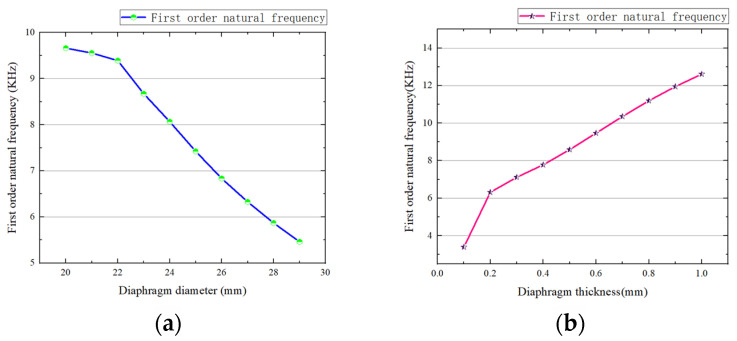
(**a**) Relationship between diaphragm diameter and the first natural frequency. (**b**) Relationship between diaphragm thickness and the first natural frequency.

In this paper, a copper alloy was selected as the raw material for processing and manufacturing, and the influence of its diameters on the frequency characteristics was studied [14]. The relationship curve between the diameter and thickness and the first natural frequency, which is the working model of the diaphragm, was obtained by applying fixed constraints as shown in Figure 4a,b, respectively.

It can be seen from Figure 4a that the previous natural frequency of the diaphragm had a negative correlation with the diameter of the diaphragm. Therefore, the size parameters were selected according to the actual working conditions. The size parameter of the diaphragm was selected as 27 mm.

## 4. Setup 3D Printing System

**Figure 5:**
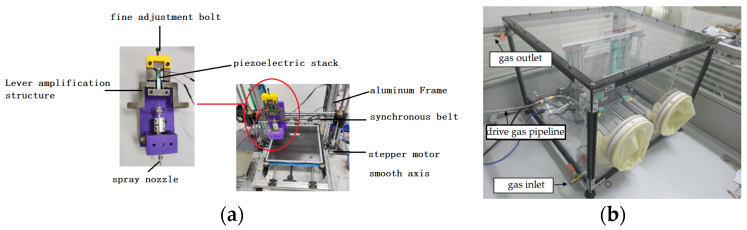
(**a**) 3D Printing system. (**b**) Sealing system.

**Figure 6:**
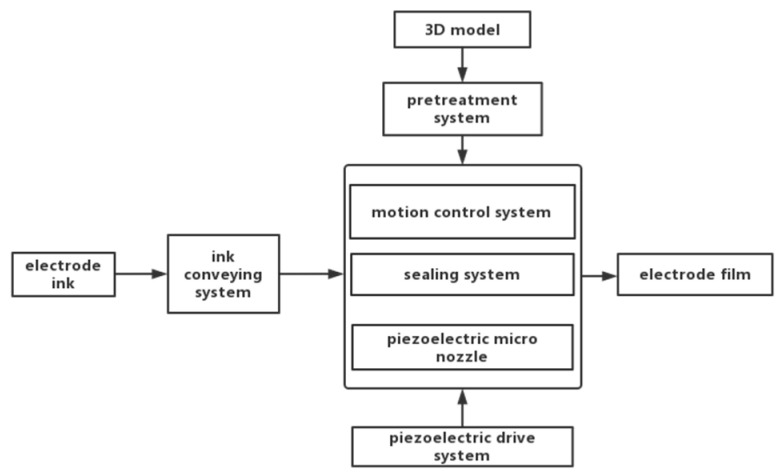
Workflow of ink-jet printing.

The printing system includes a pretreatment system, piezoelectric micro nozzle, motion control system, piezoelectric drive system, sealing system and liquid conveying system, as shown in Figure 5 [10]. Piezoelectric micronozzles are widely used in 3D printing systems. Wang L et al. designed a triangular displacement amplifier for a piezoelectric micronozzle valve based on the lever amplification principle. The magnification is seven times and the maximum output displacement is 320.6 μm [15]. Lu Shizhou et al. of the Harbin University of Technology developed a microjet valve with a piezoelectric diamond mechanism for amplification, and the stroke of the striker is close to 500 μm [16]. Hu Junfeng et al. designed a compliant mechanism with a driving force of 56.4 N and a magnification of 10.3 by combining the lever mechanism with the bridge mechanism, which can realize the reciprocating motion of the striker with a frequency of 245 Hz and a maximum output displacement of 808 μm [17]. Kuang-Chao Fan et al. presented the design, fabrication and tests of a piezoelectric-type droplet generator, which is to actuate a disk-type PZT to push the liquid out of the droplet generator and form a near-spherical droplet [18]. Lingyun Wanga proposed a noncontact jetting dispenser driven by a piezoelectric stack actuator. Its maximum jetting frequency is 65 Hz, and droplets of 1.07 mm diameter are produced by a stainless steel nozzle of 0.25 mm diameter in the experimental study [15]. In this paper, the lever amplification structure is driven by a piezoelectric stack. The displacement amplifier can avoid performance degradation of the piezoelectric actuator. The magnification reaches 26 times and satisfies the 790 μm output displacement requirement. The workflow of 3D printing electrode films is shown in Figure 6.

At first, the compressed gas is transferred to the liquid storage bottle through the electrical proportional valve, and the ink is squeezed into the liquid cavity of the spray head to remove the air in the cavity after starting the air compressor. During this process, it is necessary to ensure that the air in the spray cavity is completely discharged; otherwise, the injection effect of the micro-spray valve will be affected, and in severe cases, the ink will not be ejected. The liquid droplet is suspended at the nozzle in a semi-crescent shape by setting the voltage value of the voltage signal generator to control the output air pressure of the electrical proportional valve. Then, the waveform, voltage and frequency of the piezoelectric drive system are adjusted to output the appropriate signals. The geometric dimensions of the electrode film are designated in the 3D drawing software on the upper computer and saved in the stp format. The model is converted into G code by setting the process parameters in the 3D printing slicing software PrusaSlicer. The program is transferred to the motion control platform until the platform is heated to the given temperature. The motion control platform drives the microjet valve to move according to the trajectory set by the program. The microjet valve begins to work by the vibration of the piezoelectric stack, which is driven by the amplified signal from the driving power. During the printing process, the electrode material is cured and forms a thin film as the solvent continues to volatilize. The thickness of the electrode film is controllable, and the final required thickness can be determined by setting the number of layers to be printed and adding them layer by layer. As the volume of the microjet droplets is very small, the thickness of each of the printed layers can be within 0.1 mm.

## 5. Effection of Printing Parameters

### 5.1. Effection of Drive Signal Waveform

**Figure 7:**
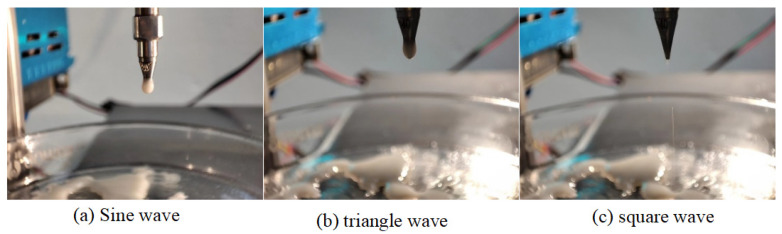
Microdroplet injection under three driving signals.

Silicon dioxide dispersion was used as the ink material, and different signals (sine wave, triangular wave and square wave) with the same signal parameters (35 Hz frequency, 3 V voltage amplitude) were inputted to drive the piezoelectric stack. The image taken is shown in Figure 7.

Neither sine waves nor triangular waves can achieve droplet injection, as shown in Figure 6. A microdroplet jet was achieved by the square wave. The piezoelectric actuator is used to control the opening and closing of the nozzle to realize a drop in demand. The speed, diameter and frequency of the jet can be controlled by adjusting the waveform parameters of the square wave to optimize the printing quality of the different ink materials.

### 5.2. Effection of Drive Waveform Amplitude and Frequency

**Figure 8:**
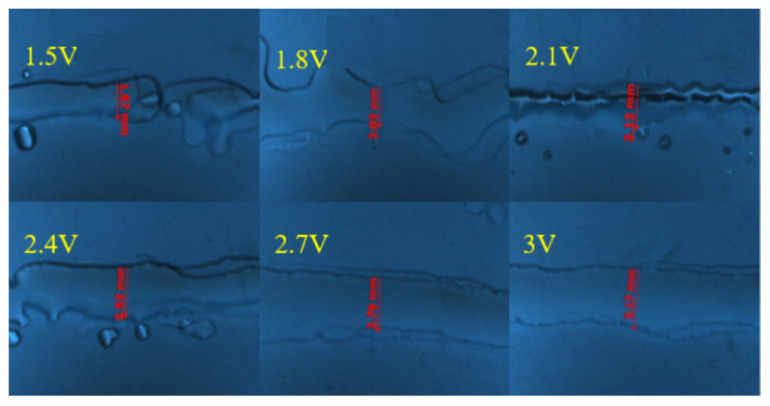
Comparison of print lines under different voltages.

**Figure 9:**
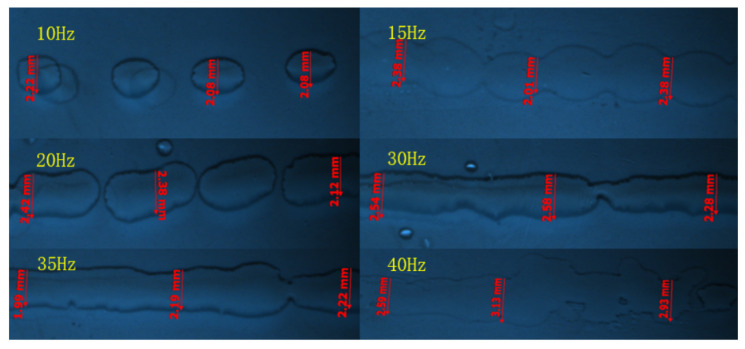
Comparison of print lines at different frequencies.

The major impact on the electrode film-forming quality is the dimensional accuracy of the printed 2D lines. The experimental conditions include 23 mm nozzle height, 0.4 mm nozzle inner diameter, 50 Hz frequency, 0.6 m/s moving speed and 5 Pa back pressure. The printing experiments are compared under the input voltages of 1.5 V, 1.8 V, 2.1 V, 2.4 V, 2.7 V and 3 V, respectively. The drive wave frequency ranges from 10 Hz to 40 Hz. The printed lines are photographed and measured using a Zeiss optical microscope (SteREO Discover V20, ZEISS, Jena, Germany). The final measurement results are shown in Figure 8 and Figure 9, respectively.

Although the printed lines are relatively thin, the quality of the printed lines is poor, and some satellite droplets appear around the lines when the voltage range from 1.5 V to 2.4 V and frequency range from 10 Hz to 20 Hz. This may be due to the relatively small amplitude of the driving voltage and frequency, resulting in relatively small pressure waves. The jet is unstable at the nozzle due to insufficient kinetic energy, and some liquid droplets are dispersed. The larger the amplitude of the driving voltage and frequency, the greater the deformation of the membrane and the larger the volume of the extruded liquid droplets, resulting in a wider line size.

### 5.3. Effection of Nozzle’s Height and Diameter

**Figure 10:**
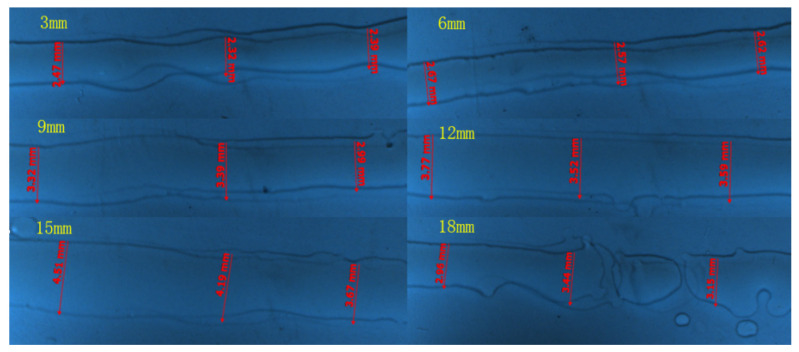
Comparison of print lines at different nozzle heights.

The nozzle height refers to the vertical distance between the nozzle end surface and the substrate to be deposited, which has a significant impact on the final drop formation of the liquid droplets. The nozzle height ranges are set as 3 mm, 6 mm, 9 mm, 12 mm and 15 mm. The other experimental conditions are as follows: nozzle inner diameter 0.4 mm, frequency 50 Hz, voltage 2.7 V, moving speed 0.6 m/s and back pressure 5 Pa. The experimental results are shown in Figure 10.

As the height of the printing head continues to increase, the width of the printing line gradually increases. The printing molding quality is good and the lines are relatively flat when the height ranges from 6 mm to 12 mm. Within 6 mm, the spray is relatively close to the substrate, the liquid droplets cannot spread well, and the printing lines are relatively distorted. The resistance encountered during the process of spraying the falling ink droplets is negligible. The higher the height, the greater the kinetic energy carried by the deposition on the substrate under the action of gravity. The larger kinetic energy is converted into more kinetic energy to disperse the droplets when the droplets collide with the substrate. Therefore, the higher the height, the larger the diameter of the dispersion, and the wider the width of the lines.

**Figure 11:**
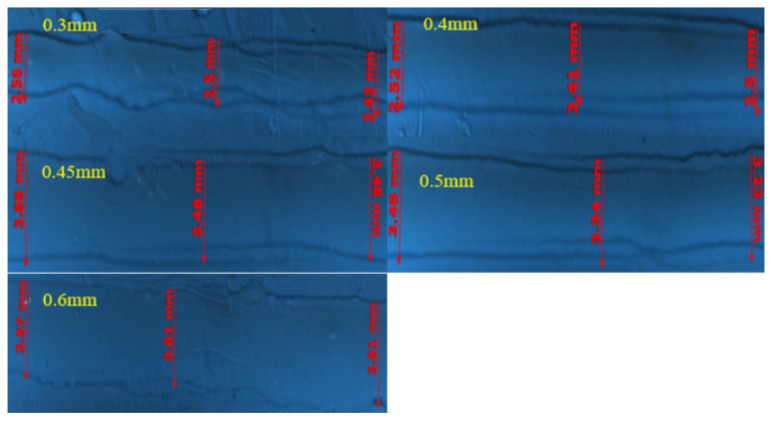
Comparison of print lines at different nozzle diameters.

The nozzle diameters in this experiment are set up as follows: 0.3 mm, 0.4 mm, 0.45 mm, 0.5 mm and 0.6 mm. The other experimental conditions are as follows: 50 Hz frequency, 2.7 V voltage, 0.6 m/s moving speed, 5 Pa back pressure and 8 mm height. The experimental results are shown in Figure 11.

The larger the nozzle diameter, the larger the printed line width, as shown in Figure 11.

## 6. Experiment with Thermal Battery Electrode Ink Printing

**Figure 12:**
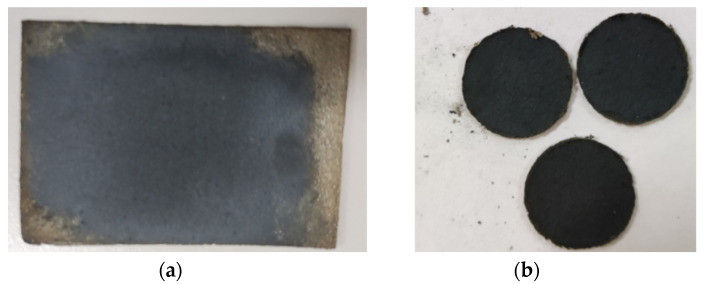
(**a**) Electrode film after preliminary drying. (**b**) Electrode film after processing.

The basic process of the 3D printing electrode film is set as follows. First, it is necessary to seal the box space for exhaust treatment to ensure a dry printing environment. Open the air inlet and inject argon gas. Close the air outlet after 5 min. Then, exhaust the nozzle through the side door of the box and put the electrode film base into the box. Inject a certain amount of argon gas until the gloves are extruded by adjusting the feed pressure. At this time, the box environment meets the printing requirements. Finally, send a printing command to the printer and input a drive signal to start printing until printing is completed. The experimental conditions are set as follows: nozzle diameter is 0.6 mm, nozzle movement speed is 90 mm/s, printing height is 8 mm, wiring width is 1mm, printing layers is 20, input voltage is 3 V and signal frequency is 35 Hz. Place the printed electrode film in a vacuum drying box and dry for 30 min at 200 °C to avoid shape deformation caused by excessive drying or wetting. The electrode film after drying is shown in Figure 12a. The electrode film begins to take shape after processing, as shown in Figure 12b. After preliminary drying, the film can effectively avoid excessive drying or wetting, which may affect the film processing effect. After the film is processed, as shown in Figure 12b, the film electrode is completely dried and assembled into a single battery for discharge testing.

**Figure 13:**
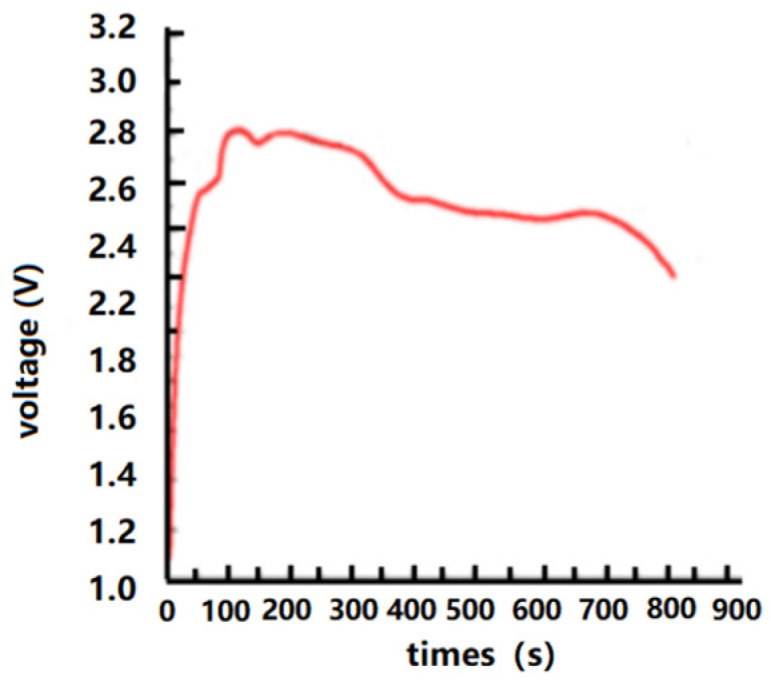
Discharge performance in a static state.

The electrical performance of the thermal batteries using electrode thin films is measured, as shown in Figure 13. The discharge performance in a static state is shown in Figure 13.

As shown in Figure 13, the thermal batteries are activated in a high-temperature environment as the temperature increases in the tube furnace. The measured static voltage increases sharply within 50 s, and the increasing trend slows down after one minute. However, the voltage reaches its peak and tends to flatten out at around 100 s. The stabilized voltage is approximately 2.62 V. There is no significant voltage fluctuation in the resting curve after 100 s, which indicates that the internal components of the thin film prepared are very stable and reliable.

## 7. Conclusions

In order to ensure the performance of the thermal battery electrode, a 3D printing device based on the direct drive of a piezoelectric actuator, with optimized parameters, is proposed in this work. The complete spraying process of the liquid in the cavity from the nozzle to form tiny droplets is simulated. The spray performance of the nozzle cavities with conical sections is optimal compared to those with semicircle and rectangular cross sections. Compared to sine waves and triangular waves, only square waves can achieve droplet injection. The drive voltage and frequency are also investigated. The larger amplitude of the driving voltage and frequency results in a wider line size due to the greater deformation of the membrane and larger volume of the extruded liquid droplets. The printing performance at different nozzle heights and diameters is compared to obtain the optimized parameters. The width of the printing line increases with the height of the printing head. This is because the higher kinetic energy makes the droplets bigger when they collide with the substrate. The viscosity range of the ink is limited to 25–50 cps in order to obtain reasonable film thickness successfully. The prototype of the whole 3D printing system with the printing process is set up. The film sample is printed and assembled on a thermal battery with optimized parameters and testifies that the printing performance meets the requirements. The film proved to be very stable in the discharge experiment.
